# Identification and Active Evaluation of Antioxidant Peptides from Protein Hydrolysates of Skipjack Tuna (*Katsuwonus pelamis*) Head

**DOI:** 10.3390/antiox8080318

**Published:** 2019-08-19

**Authors:** Lun Zhang, Guo-Xu Zhao, Yu-Qin Zhao, Yi-Ting Qiu, Chang-Feng Chi, Bin Wang

**Affiliations:** 1 Zhejiang Provincial Engineering Technology Research Center of Marine Biomedical Products, School of Food and Pharmacy, Zhejiang Ocean University, Zhoushan 316022, China; 2 National and Provincial Joint Laboratory of Exploration and Utilization of Marine Aquatic Genetic Resources, National Engineering Research Center of Marine Facilities Aquaculture, School of Marine Science and Technology, Zhejiang Ocean University, Zhoushan 316022, China

**Keywords:** skipjack tuna (*Katsuwonus pelamis*), head, peptide, antioxidant activity, stability

## Abstract

For the full use of fish by-products to produce antioxidant peptides, skipjack tuna (*Katsuwonus pelamis*) heads generated during can processing were defatted and hydrolyzed using the in vitro gastrointestinal (GI) digestion (pepsin–trypsin system) method and six antioxidant peptides (P1 to P6) were purified from the head hydrolysate (KPH) using ultrafiltration and serial chromatography methods. Six isolated peptides (P1 to P6) were identified as Val-Glu-Glu (VEE, P1), Trp-Met-Phe-Asp-Trp (WMFDW, P2), Asp-Ala-Gly-Pro-Tyr-Gly-Pro-Ile (DAGPYGPI, P3), Trp-Met-Gly-Pro-Tyr (WMGPY, P4), Glu-Arg-Gly-Pro-Leu-Gly-Pro-His (ERGPLGPH, P5), and Glu-Met- Gly-Pro-Ala (EMGPA, P6), respectively, using a protein sequencer and electrospray ionization-mass spectrometer. Among skipjack tuna head hydrolysates, fractions, and six isolated peptides (P1 to P6), WMFDW (P2), WMGPY (P4), and EMGPA (P6) showed the highest radical scavenging activities on 2,2-diphenyl-1-picrylhydrazyl (DPPH) (EC_50_ values of 0.31, 0.33, and 0.46 mg/mL for WMFDW, WMGPY, and EMGPA, respectively), hydroxyl (EC_50_ values of 0.30, 0.43, and 0.52 mg/mL for WMFDW, WMGPY, and EMGPA, respectively), and superoxide anion (EC_50_ values of 0.56, 0.38, and 0.71 mg/mL for WMFDW, WMGPY, and EMGPA, respectively). Moreover, WMFDW, WMGPY, and EMGPA showed strong capability in reducing power and lipd peroxidation inhibition in the linoleic acid system. In addition, WMFDW, WMGPY, and EMGPA can retain strong antioxidant activity at temperatures lower than 60 °C and pH values ranged from 5 to 9. The results showed that six isolated peptides (P1 to P6) from skipjack tuna heads, especially WMFDW, WMGPY, and EMGPA, might be applied in health care products acting as powerful antioxidant agents.

## 1. Introduction

Bioactive peptides generally range in molecular size from 2 to 20 amino acid residues and show a large number of potential physiologic functions within biological bodies, such as antioxidant, opioid, antihypertensive, immunomodulatory, antithrombotic, and antibacterial activities [1,2,3,4]. The identification, quantitation and activity evaluation of food-derived bioactive peptides are very important for the characterization of its production, biological value and benefits for consumer health [5,6]. In general, bioactive peptides can be produced during food manufacturing either by the spontaneous hydrolysis of food proteins with endogenous digestive enzymes, or by designing hydrolysis conditions with commercial proteases [7,8,9]. In addition, bioactive peptides can also be released during the gastrointestinal digestion of the ingested foods [10]. 

Presently, a large number of bioactive peptides has been produced from food proteins of different origins like milk, eggs, soya, fish, mollusk and meat [7,11,12,13]. Among them, antioxidant peptides (APs) derived from aquatics and their byproducts, such as the heads and viscera of sardinella [14], skin and viscera of cuttlefish [15], carcass and viscera of Nile tilapia [16], heads and skins of bluefin leatherjacket (*Navodon septentrionalis*) [6,12], and swim bladders and muscle of miiuy croaker (*Miichthys miiuy*) [8,17], have attracted much investigative and public interests because they can positively influence health in the body and have huge potential applied in functional and health products [5,18]. Two hexapeptides of WAFAPA and MYPGLA from the protein hydrolysate of blue-spotted stingray can protect DNA and proteins from oxidation and show stronger capability to restrain H_2_O_2_-induced lipid oxidation than carnosine [19]. The gelatin hydrolysate fraction of Nile tilapia skin (FSGHFs) (molecular weight (MW) < 1000 Da) can increase cell viability and membrane integrity, suppress reactive oxide species (ROS) production and epithelial barrier damage in H_2_O_2_-induced intestinal porcine epithelial cells (IPEC-J2) by stimulating the nuclear factor erythroid 2-related factor 2 (Nrf2) to increase the level of glutathione and the mRNA and protein expression of catalytic and modulatory subunits of γ-glutamyl cysteine ligase [20]. In addition, a pentapeptide of YGDEY from tilapia gelatin hydrolysates can inhibit ultraviolet B (UVB)-mediated skin photoaging by decreased MMP-1 (collagenase) and MMP-9 (gelatinase) expression, increase antioxidant factors superoxide dismutase (SOD) and glutathione (GSH) expression, and prevent DNA from oxidative damage [21]. Tao et al. reported that GAERP, GEREANVM, and AEVG can effectively scavenge free radicals and protect human liver cancer cell line (HepG2) from H_2_O_2_-induced oxidative damage through reducing the malondialdehyde (MDA) level and promoting the contents of glutathione reductase (GSH-Rx), SOD, glutathione peroxidase (GSH-Px), and catalase (CAT) [22]. The literature proved that seafood-derived APs have high antioxidant capability and can be applied in health care food to protect cells and organisms against oxidative stress.

As an epipelagic oceanic species, skipjack tuna (*Katsuwonus pelamis*) mainly live in tropical and subtropical seas with over 2 million metric tons of production every year in the Pacific Ocean [23]. In China, approximately 200,000 tons of tuna are processed every year and its byproducts accounted for approximately 50% of the raw material, including fish bones, head, scales, viscera and skins, are generated during tuna can processing [24]. Traditionally, these tune byproducts are processed into low-value fish flour utilized in animal feeds or dumped as harmful wastes [24]. In recent years, the production of bioactive molecules including collagen, gelatin, and bioactive peptides using tuna byproducts were studied as a promising means to increasing tuna value and lowering environmental pollution. Yu et al. prepared collagens from the spines and skulls of skipjack tuna and characterized them as type I collagen [24]. Shyni et al. prepared the skin gelatin of skipjack tuna with yield of 11.3% and analyzed its physical and chemical properties, such as MW, viscosity, odor, color, melting point, amino acid composition, and water-holding capacity [25]. GADIVA and GAEGFIF from the gelatin hydrolysate of skipjack tuna bone exhibited high inhibiting ability on lipid peroxidation and strong radical scavenging activities on 2,2-diphenyl-1-picrylhydrazyl (DPPH), hydroxyl, superoxide anion, and 2,2’-azino-bis-3-ethylbenzothiazoline-6-sulfonic acid (ABTS). They attributed the high activity of GADIVA and GAEGFIF to small MW and hydrophobic/aromatic amino acids [10]. Chi et al. prepared alcalase and neutrase hydrolysates of skipjack tuna dark muscle and illuminated the effects of amino acid compositions, MW, and AP profiles on their protective capacities of lipid peroxidation [23]. However, no literature on APs from skipjack tuna head was found. Therefore, the objectives of this investigate were to prepare APs from the head hydrolysate of skipjack tuna, identify the MWs and amino acid sequences of isolated APs, and assess their in vitro antioxidant activities at the end of this study. 

## 2. Materials and Methods

### 2.1. Materials

Skipjack tuna (*K. pelamis*) heads were provided by Ningbo Todayfood Co. Ltd. (China). Diethylethanolamine (DEAE)-52 cellulose and Sephadex G-25 were purchased from Shanghai Source Poly Biological Technology Co., Ltd. (China). Trifluoroacetic acid (TFA) and Acetonitrile (ACN) of liquid chromatogram grade were purchased from Thermo Fisher Scientific Co., Ltd. (Shanghai, China). DPPH and GSH of pharmaceutical secondary standard were purchased from Sigma-Aldrich Trading Co., Ltd. (Shanghai, China). Val-Glu-Glu (VEE, P1), Trp-Met-Phe-Asp-Trp (WMFDW, P2), Asp-Ala-Gly-Pro-Tyr-Gly-Pro-Ile (DAGPYGPI, P3), Trp-Met-Gly-Pro-Tyr (WMGPY, P4), Glu-Arg-Gly-Pro-Leu-Gly-Pro-His (ERGPLGPH, P5), and Glu-Met- Gly-Pro-Ala (EMGPA, P6) were synthesized in China Peptides Co. (Suzhou, China).

### 2.2. Preparation of the Protein Hydrolysate (KPH) of Kipjack Tuna Heads

Skipjack tuna heads were thawed, cleaned with tap water, minced, and defatted with the previously mentioned method [6]. In brief, the homogenate was mixed with isopropanol at a ratio of 1:5 (*w/v*) and left to stand at 30 ± 2 °C with continuous agitation, and the isopropanol was changed after 2.0 h. Finally, the solution was centrifuged at 6000 *g* for 15 min, and the precipitate was dried at 35 ± 2 °C.

The protein hydrolysate (KPH) of skipjack tuna heads was prepared using the in vitro gastrointestinal (GI) digestion method [10]. Briefly, the defatted powders dispersed in distilled water (DW) (pH 1.5, 1%) were firstly hydrolyzed by pepsin with a protease dosage of 1 g pepsin/100 g defatted powder under the conditions of 37.0 ± 2 °C and pH 1.5. Two hours later, the pH of the degraded solution was adjusted to 7.0 using 1.0 M NaOH solution and further hydrolyzed using trypsin with a protease dosage of 1 g trypsin/100 g defatted powder. After 2 h, the temperature of the solution was increased to 95 ± 2 °C and kept for 10 min. Finally, the solution was centrifuged at 9000 *g* for 10 min at 4 °C and the supernatant (KPH) was lyophilized and stored in a −20 °C freezer. 

The concentrations of KPH and its fractions were measured by the method described by Bradford [26] and represented as mg protein/mL. The degree of the hydrolysis (DH) analysis of protein hydrolysates was performed according to the method described by Yang et al. [13].

### 2.3. Preparation of APs from KPH

#### 2.3.1. Ultrafiltration

KPH was ultra-filtrated using MW cut-off (MWCO) membranes of 3, 5 and 10 kDa, and four resulting fractions, defined as UF-1 (MW < 3 kDa), UF-2 (3–5 kDa), UF-3 (5–10 kDa), and UF-4 (MW > 10 kDa), were prepared and lyophilized.

#### 2.3.2. Separation of APs by Chromatography Methods

The separation process of APs from UF-1 was performed according to the method described by Yang et al. [13]. Briefly, UF-1 (5 mL, 40.0 mg protein/mL) was injected into a pre-equilibrated DEAE-52 cellulose column (2.6 cm × 120 cm) and eluted with 150 mL DW and 0.1, 0.5, and 1.0 M NaCl solution at a flow rate of 1.0 mL/min. Each eluted fraction (5 mL) was collected and monitored at 214 nm. Finally, six fractions (defined as AC-1 to AC-6) were prepared on the peaks of the 214-nm chromatographic curve, and 5 mL AC-4 solution (20.0 mg protein/mL) was added into a Sephadex G-25 column (2.6 cm × 160 cm) and separated with a flow rate of 0.8 mL/min. Three active factions (defined as GC-1, GC-2, and GC-3) were prepared on the chromatographic curve on the 214-nm absorbance of each eluate (3 mL). Finally, GC-3 (20 μL, 10.0 mg protein/mL) was further purified by a High-performance liquid chromatography (HPLC) column of Zorbax, SB C-18 (4.6 mm × 250 mm) on an Agilent 1260 system (Agilent Ltd., Santa Rosa, CA, USA). GC-3 was eluted with ACN solution containing 0.1% TFA at 1.0 mL/min and the ACN concentration increased from 0 to 50% in 25 min in a linear gradient manner. Six APs (P1 to P6) were prepared on the HPLC chromatographic peaks at 214 nm.

### 2.4. Analysis of Amino Acid Sequence and MW

The amino acid sequences of six APs (P1 to P6) were analyzed using a protein sequencer of Applied Biosystems 494 (Applied Biosystems Inc, Foster City, CA, USA). The MWs of six APs (P1 to P6) were determined by a quadrupole time-of-flight mass spectrometer (MS) coupled with an electrospray ionization (ESI) source, respectively. 

### 2.5. Antioxidant Activity

The radical scavenging assays of six APs (P1 to P6) were tested on the method described by Yang et al. [13]. The half elimination ratio (EC_50_) was used to express the radical scavenging capability of six APs and defined as the concentration of a sample that caused a 50% decrease in the initial radical concentration. The assays of lipid peroxidation inhibition and reducing power were determined by the method described by He et al. [17]. 

### 2.6. Stability Properties

The stability of APs (P2, P4, and P6) was measured according to the previous method [13]. The thermostability of APs was determined using a water bath for 0.5 h and the temperature was set as 20, 40, 60, 80, or 100 °C. The influences of pH treatments at pH values of 3, 5, 7, 9, or 11 were used to evaluate the pH stability of APs (P2, P4, and P6) at 25 °C for 2.5 h. The EC_50_ values of APs (P2, P4, and P6) on hydroxyl radical were used as an index and tested using the methods described in Section 2.5.

### 2.7. Statistical Analysis

The data are expressed as the mean ± standard deviation (SD, *n* = 3). An ANOVA test was used to analyze the differences in data from different groups, and a *p*-value of less than 0.05 was considered statistically significant.

## 3. Results and Discussion

### 3.1. Preparation of the Protein Hydrolysate (KPH) of Kipjack Tuna Heads

According to the chemical composition analysis, the major components in skipjack tuna heads were moisture, protein, fat, and ash, with contents of 67.1 ± 3.4, 14.6 ± 0.86, 10.2 ± 0.25, and 6.2 ± 0.93 g/100 g, respectively, on the wet weight basis. The data demonstrated that skipjack tuna heads were suitable for the production of bioactive peptides due to their high protein content.

Proteins of skipjack tuna heads were hydrolyzed by the in vitro GI digestion system, and the prepared hydrolysate (KPH) with a DH of 25.76 ± 1.68% can effectively scavenge DPPH radical with an EC_50_ value of 5.36 mg protein/mL. The EC_50_ value was lower than those of protein hydrolysates from skate cartilage (13.13 mg protein/mL) [3] and bluefin leatherjacket skin (5.23 mg protein/mL) [12], but higher than those of protein hydrolysates from *Tergillarca granosa* muscle (3.55 mg protein/mL) [13], tilapia skin (3.66 mg/mL) [16], thornback ray (1.98 mg/mL) [27], and salmon pectoral fin (1.63 mg/mL) [28]. Therefore, the results indicated that the in vitro GI digestion model was effective in preparing bioactive peptides of skipjack tuna heads.

### 3.2. Purification of APs from KPH

#### 3.2.1. Ultrafiltration

**Figure 1:**
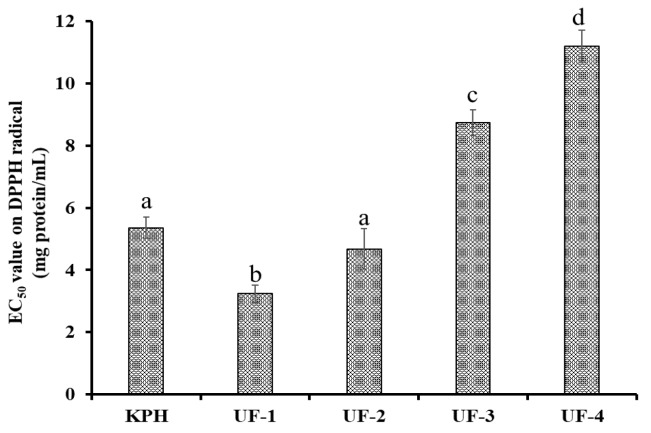
The half elimination ratio (EC_50_) of the hydrolysate (KPH) and its four active fractions (defined as UF-1 to UF-4) by ultrafiltration on the 2,2-diphenyl-1-picrylhydrazyl (DPPH) radical. The data are presented as the mean ± SD (*n* = 3). ^a–d^ The same superscripts indicate no significant difference (*p* > 0.05).

KPH was divided by ultrafiltration with MWCO membranes of 3, 5, and 10 kDa, and four fractions, namely, UF-1 (MW < 3 kDa), UF-2 (3–5 kDa), UF-3 (5–10 kDa), and UF-4 (MW > 10 kDa), were prepared, and their scavenging activity on the DPPH radical is shown in Figure 1. The EC_50_ value of UF-1 was 3.24 mg protein/mL, which was significantly stronger than those of KPH (5.36 mg protein/mL), and other three fractions (EC_50_ value of 4.68, 8.74, and 11.20 mg protein/mL for UF-2, UF-3, and UF-4, respectively) (*p < 0.05*). KPH consisted of a wide variety of APs with different molecular sizes. Li et al. reported that hydrolysate fractions with a lower MW were more accessible to free radicals and showed high radical scavenging activity [29]. Therefore, UF-1 was rich in low MW APs and selected for the subsequent chromatographic separation.

#### 3.2.2. Anion-Exchange Chromatography

**Figure 2:**
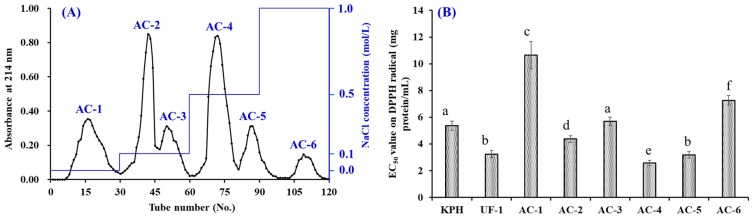
Elution diagram of UF-I in the diethylethanolamine (DEAE)-52 cellulose column of (**A**) and EC_50_ values of UF-I and its active fractions (defined as AC-1 to AC-6) on the DPPH radical (**B**). The data are presented as the mean ± SD (*n* = 3). ^a–f^ The same superscripts indicate no significant difference (*p* > 0.05).

Figure 2A showed the anion-exchange chromatographic chart of six fractions (AC-1 to AC-6) from UF-1. According to their adsorption capacity with the DEAE-52 cellulose, AC-1, AC-2 and AC-3, AC-4 and AC-5, and AC-6 were gradually eluted out by an eluent of DW, 0.1 NaCl solution, 0.5 NaCl solution, and 1.0 M NaCl solution, respectively. 

The data in Figure 2B suggested that the EC_50_ value of AC-4 (2.58 mg protein/mL) was significantly less than those of UF-I (3.24 mg protein/mL) and other five fractions (EC_50_ values of 10.65, 4.37, 5.69, 3.18, and 7.26 mg protein/mL for AC-1, AC-2, AC-3, AC-5, and AC-6, respectively) (*p* < 0.05). Amino acid residues in bioactive peptides with the acidic and hydrophobic groups can be easily adsorbed to the anion-exchange resin (Q Sepharose FF, DEAE, DEAE-52 cellulose and XK 260) and separated from other peptides [6,30]. Moreover, amino acid residues with acidic and/or hydrophobic groups, such as Glu, Met, His, Asp and Pro, are recognized as one of the vital factors for the activity of APs [3,31]. Thus, AC-4 isolated should contain APs with high potential activity and was selected for the following experiment.

#### 3.2.3. Gel Filtration Chromatography (GFC)

**Figure 3:**
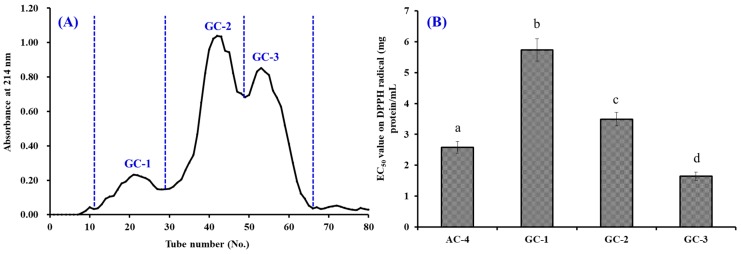
Elution diagram of AC-4 in the Sephadex G-25 column of (**A**) and EC_50_ value of AC-4 and its three active fractions (defined as GC-1 to GC-3) on the DPPH radical (**B**). The data are presented as the mean ± SD (*n* = 3). ^a–d^ The same superscripts indicate no significant difference (*p* > 0.05).

Figure 3A indicated that AC-4 was separated into three active components (GC-1 to GC-3) by the Sephadex G-25 column. The DPPH radical scavenging assay showed that GC-3 with an EC_50_ value of 1.65 mg protein/mL had a significantly stronger activity than AC-4 (2.58 mg protein/mL), and the other two active components (EC_50_ values of 5.73 and 3.49 mg protein/mL for GC-1 and GC-2, respectively) (*p* < 0.05). 

Gel filtration has been used for separating bioactive peptides from protein hydrolysates of aquatic byproducts, such as salmon [28], sardinelle [14], cartilaginous fish [32,33,34], blue mussel [31], skipjack tuna [23], miiuy croaker [8], and cuttlefish [15]. The presented results were in agreement with the report by Li et al. and Pan et al. that peptide components with a low–average MW possess stronger antioxidant activities than their larger counterparts [29,35]. Therefore, GC-3 was suitable for the following purification process. 

#### 3.2.4. Purification of APs from GC-3 by Reversed-Phase HPLC (RP-HPLC)

**Figure 4:**
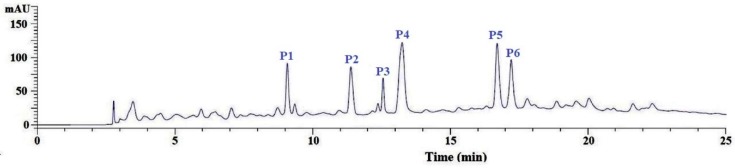
Reversed-phase high-performance liquid chromatography (RP-HPLC) chromatogram of GC-3 using a Zorbax, SB C-18 column (4.6 mm × 250 mm).

Finally, GC-3 was purified using a Zorbax C-18 column on an Agilent 1260 HPLC system and the elution profile is shown in Figure 4. The eluted APs were collected on their chromatographic peaks. In the end, six APs from GC-3, with a retention time (RT) of 9.05 min (P1), 11.36 min (P2), 12.63 min (P3), 13.25 min (P4), 16.78 min (P5), and 17.22 min (P6), were prepared for molecular structural identification and bioactivity analysis. 

### 3.3. Molecular Structural Analysis

**Figure 5:**
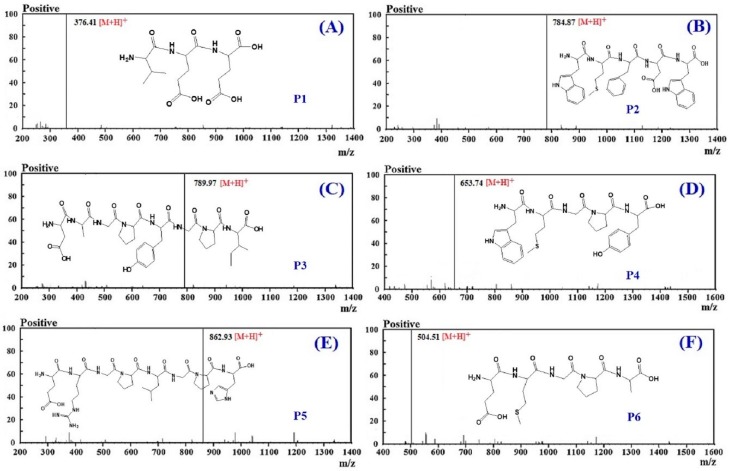
Mass spectrogram of P1 (**A**), P2 (**B**), P3 (**C**), P4 (**D**), P5 (**E**), and P6 (**F**) from the hydrolysate of skipjack tuna (*K. pelamis*) heads.

The amino acid sequences of the six isolated APs (P1 to P6) were analyzed by a protein sequencer and identified as Val-Glu-Glu (VEE, P1), Trp-Met-Phe-Asp-Trp (WMFDW, P2), Asp-Ala-Gly-Pro-Tyr-Gly-Pro-Ile (DAGPYGPI, P3), Trp-Met-Gly-Pro-Tyr (WMGPY, P4), Glu-Arg-Gly-Pro-Leu-Gly-Pro-His (ERGPLGPH, P5), and Glu-Met- Gly-Pro-Ala (EMGPA, P6), respectively (Table 1). In addition, the MWs of VEE (P1), WMFDW (P2), DAGPYGPI (P3), WMGPY (P4), ERGPLGPH (P5), and EMGPA (P6) were 375.39, 783.90, 788.85, 652.79, 861.95, and 503.58 Da, respectively (Figure 5). Those MW data of the six isolated APs (P1 to P6) agreed well with their theoretical masses (Table 1).

### 3.4. Antioxidant Activity

**Figure 6:**
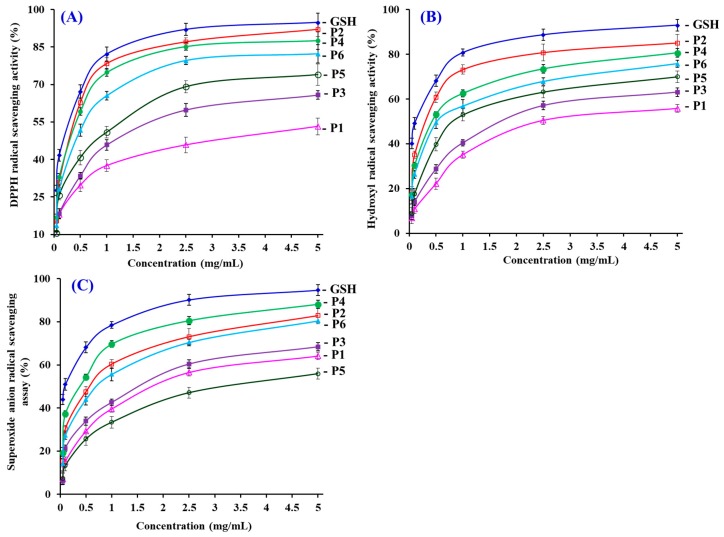
DPPH radical (**A**), hydroxyl radical (**B**), and superoxide anion radical (**C**) scavenging activities of six isolated APs (P1 to P6) from the hydrolysate of skipjack tuna (*K. pelamis*) heads. The data are presented as the mean ± SD (*n* = 3).

**Figure 7:**
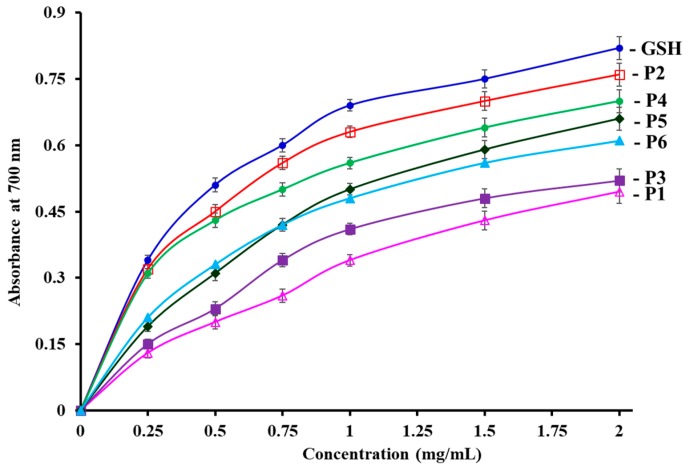
The reducing power of six isolated APs (P1 to P6) from the hydrolysate of skipjack tuna (*K. pelamis*) heads. The data are presented as the mean ± SD (*n* = 3).

**Figure 8:**
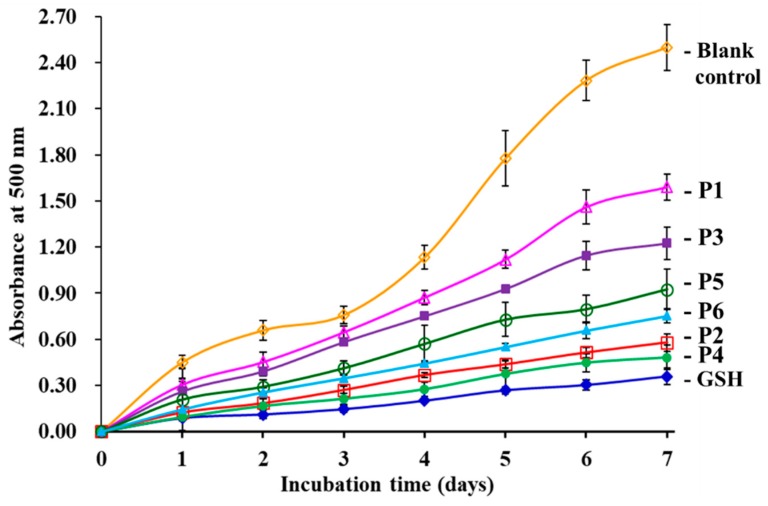
Lipid peroxidation inhibition capability of six isolated APs (P1 to P6) from the hydrolysate of skipjack tuna (*K. pelamis*) heads. The data are presented as the mean ± SD (*n* = 3).

In the experiment, radical scavenging, reducing power and lipid peroxidation inhibiting assays were used to evaluate the activity of six isolated APs (P1 to P6) from the hydrolysate of skipjack tuna heads, and the experimental data were showed in Table 2 and Figure 6, Figure 7 and Figure 8.

#### 3.4.1. Radical Scavenging Activity

##### DPPH Radical Scavenging Activity

Figure 6A indicated that six isolated APs (P1 to P6) can positively influence the DPPH radical scavenging rates when the concentrations of APs ranged from 0.05 to 5.0 mg/mL. Among six isolated APs, the EC_50_ value of P2 (0.31 mg/mL), P4 (0.33 mg/mL), P5 (0.93 mg/mL)), and P6 (0.46 mg/mL) was less than 1.0 mg/mL (Table 2). Moreover, the EC_50_ value of P2 and P4 did not show a significant difference with the positive control of GSH (*p* > 0.05). The EC_50_ values of P2 and P4 were lower than those of most APs from protein hydrolysates of aquatics and their processing by-products, such as blue mussel (*M. edulis*) (YPPAK: 2.62 mg/mL) [31], skate cartilages (FIMGPY: 2.60 mg/mL; GPAGDY: 3.48 mg/mL; IVAGPQ: 3.93 mg/mL) [3], the pectoral fin of salmon (TTANIEDRR: 2.50 mg/mL) [36], spanish mackerel skins (PFGPD: 0.80 mg/mL; PYGAKG: 3.02mg/mL; YGPM: 0.72 mg/mL) [37], *Tergillarca granosa* muscle (MDLFTE: 0.53 mg/mL; WPPD: 0.36 mg/mL) [34], miiuy croaker swim bladders (FPYLRH: 0.78 mg/mL; GIEWA: 0.78 mg/mL) [8], croceine croaker muscle (YLMSR: 1.35 mg/mL) [38], and loach (PSYV: 17.0 mg/mL) [39]. Nevertheless, the EC_50_ values of P2 and P4 were greater than those of APs from the hydrolysates of grass carp skin (HFGBPFH: 0.20 mg/mL) [40], bluefin leatherjacket skin (FIGP: 0.12 mg/mL) [12], and skate muscle (NWDMEKIWD: 0.29 mg/mL) [41]. Then, six isolated APs (P1 to P6), especially P2 and P4, have a strong ability to inhibit the DPPH radical reaction through donating hydrogens or scavenging free radicals.

##### Hydroxyl Radical Scavenging Activity

Figure 6B revealed that P1, P2, P3, P4, P5, and P6 can concentration-dependently scavenge hydroxyl radicals with EC_50_ values of 2.43, 0.30, 1.71, 0.43, 0.81, and 0.52 mg/mL, respectively (Table 2). P2 revealed the highest radical scavenging ability among six isolated APs, but its EC_50_ value was none the less significantly less than that of GSH (0.12 mg/mL) (*p* < 0.05). All the more important, the EC_50_ value of P2 was lower than those of most APs from hydrolysates of the muscle of croceine croaker (YLMSR: 0.35 mg/mL) [38], miiuy croaker swim bladders (GFYAA: 2.35 mg/mL; FSGLR: 2.45 mg/mL) [8], weatherfish loach (PSYV: 2.64 mg/mL) [39], conger eel (LGLNGDDVN: 0.69 mg/mL) [42], bluefin leatherjacket (GPP: 2.36 mg/mL) [6], giant squid (NADFGLNGLEGLA: 0.61 mg/mL) [43], hairtail muscle (IYG: 2.50 mg/mL; AKG: 2.38 mg/mL; KA: 1.74 mg/mL) [44], and grass carp skin (PYSFK: 2.28 mg/mL; VGGRP: 2.06 mg/mL) [40]. Nevertheless, the EC_50_ value of P2 was still greater than those of APs from bluefin leatherjacket skin (FIGP: 0.07 mg/mL) [12], *Sphyrna lewini* muscle (SAP: 0.17 mg/mL; PYFNA: 0.24 mg/mL) [32,33], and monkfish muscle (EWPAQ: 0.27 mg/mL; FLHRP: 0.11 mg/mL; LMGQW: 0.04 mg/mL) [45]. In the body, hydroxyl radicals can give rise to the process of oxidative stress to unselectively attack and oxidize biomacromolecules, and further cause some chronic diseases. The results indicated that P2 can serve as a hydroxyl radical eliminator to eliminate its injury in biological systems.

##### Superoxide Anion Radical Scavenging Assay

Figure 6C indicated that the scavenging ability of six isolated APs (P1 to P6) on the superoxide anion radical enhanced gradually following the increase in peptide content within the range of 0.05–5.0 mg/mL. The EC_50_ value of P4 was 0.38 mg/mL, which was significantly less than those of other five isolated APs (EC_50_ values of 1.79, 0.56, 1.51, 3.04, and 0.71 mg/mL for P1, P2, P3, P5, and P6, respectively), but significantly greater than that of GSH (0.09 mg/mL) (*p* < 0.05). What is more, the EC_50_ value of P4 was lower than those of APs from hydrolysates of muscle of monkfish (EWPAQ: 0.62 mg/mL) [45], skate cartilage (FIMGPY: 1.61 mg/mL; GPAGDY: 1.66 mg/mL; IVAGPQ: 1.82 mg/mL) [3], hairtail muscle (EC_50_ values of 1.34, 2.54, and 2.08 mg/mL for IYG, AKG, and KA, respectively) [44], swim bladders of miiuy croaker (EC_50_ values of 3.35, 1.92, 0.87, 3.03, and 3.04 mg/mL for FSGLR, FYKWP, GFEPY, GFYAA, and FTGMD, respectively) [8], *Tergillarca granosa* muscle (MDLFTE: 0.75 mg/mL; WPPD 0.46 mg/mL) [13], and croceine croaker muscle (MILMR: 0.993 mg/mL) [38]. However, the EC_50_ values of P4 were higher than those of APs from protein hydrolysates of round scad (HEKVC: 0.235 mg/mL; HDHPVC: 0.265 mg/mL) [46], bluefin leatherjacket skin (FIGP: 0.311 mg/mL) [12], red stingray cartilages (IEPH: 0.17 mg/mL; IEEEQ: 0.16 mg/mL) [37], and muscle of monkfish (FLHRP: 0.101 mg/mL) [45]. As a major product of the primary oxidase sources of ROS in organisms, superoxide anion radicals can damage the carbonyl compounds and initiate lipid peroxidation when they convert to the highly reactive hydroxyl radical. In addition, the potential damage of superoxide anion radicals can be scavenged by SOD. Therefore, six isolated APs (P1 to P6), especially P4, can act as the radical eliminators to eliminate the radical damage along with SOD.

#### 3.4.2. Reducing power

Figure 7 shows that six isolated APs (P1 to P6) showed concentration-dependent reducing power when AP concentrations were increased from 0.05 to 2.0 mg/mL. P2 have the highest reducing capacity on converting Fe^3+^/ferricyanide complex into the ferrous form among the six APs. However, the reducing power of the APs was lower than that of the positive control of GPS. Reducing power is referred to as the ability of antioxidants to donate electrons and hydrogen in reduction reactions in organisms. The reducing capacity of an AP is considered as a key indicator of its potential antioxidant activity [17,29]. The present results suggested that P4 can serve as primary and secondary antioxidants to decrease the oxidized intermediates of lipid peroxidation reactions through acting as an electron donor. 

#### 3.4.3. Lipid Peroxidation Inhibition Assay

Using the linoleic acid model system, the lipid peroxidation inhibition abilities of six isolated APs (P1 to P6) were determined and the lower absorbance at 700 nm illustrates higher antioxidant activity [32]. As showed in Figure 8, the absorbance value of the blank control was significantly higher than those of the six isolated APs (P1 to P6) and the positive control of GSH groups, which indicated that six isolated APs (P1 to P6) and GSH can inhibit the peroxidation of linoleic acid during 7 days incubation. In addition, the absorbance values of P2 and P4 groups was lower than those of the other four APs (P1, P3, P5 and P6). The results indicated that P2 and P4 could serve as the primary and secondary antioxidants to inhibit lipid oxidation in food products. 

### 3.5. Effects of Thermal and pH Treatments on the Stability of P2, P4 and P6

**Figure 9:**
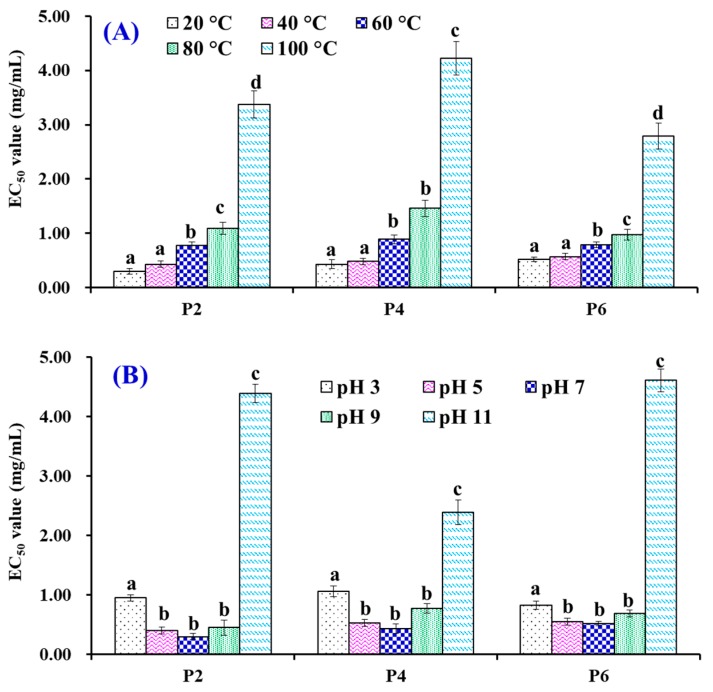
EC_50_ values of P2, P4 and P6 on hydroxyl radical scavenging activity when treated at different temperatures (**A**) and pH values (**B**). The data are expressed as the mean ± SD (*n* = 3). ^a–d^ Values with same letters of same sample indicate no significant difference (*p* > 0.05).

Figure 9A indicated that the scavenging activity of P2, P4 and P6 on hydroxyl radicals (presented as EC_50_ value) has the same varying tendency. Heat treatments can greatly affect their activity and the EC_50_ values of P2, P4, or P6 treated at 20 and 40 °C were significantly (*p* > 0.05) different from those treated at 60, 80, and 100 °C, respectively (*p* < 0.05). On the other hand, there was no significant difference in their EC_50_ values on hydroxyl radicals when P2, P4, or P6 were disposed at 20 and 40 °C for 0.5 h, respectively (*p* > 0.05).

Figure 9B shows the influences of acid and alkali treatments on P2, P4 and P6. The results indicated that the EC_50_ values of P2, P4, or P6 showed no significant difference when the pH value ranged from 5 to 9, but they were significantly different from the EC_50_ values of P2, P4, or P6 treated at pH 3 or pH 11. In addition, the alkaline treatment had a stronger effect on P2 and P6 compared with P4. 

## 4. Discussion

Proteins in seafoods and their by-products have a high structural diversity and represent a massive resource for the mining of bioactive peptides [1]. Bioactive peptides are released from the sequence of proteins by different hydrolysis methods, such as solvent extraction, microbial fermentation, enzymatic hydrolysis and chemical treatment [5]. Nevertheless, the proteolytic hydrolysis process is the popular method in health care food industries to avoid toxic chemicals or residual organic solvents in the final products. In addition, the specificity and parameters of the proteases significantly affect the MW and amino acid composition of bioactive peptides [47,48,49]. In some cases, specific enzyme combinations are used to enhance the DH of protein and prepare hydrolysates enriched with low MW APs [44,48,50].

The structural activity specialties can provide guides to speculate the activity of isolated APs. The amino acid composition and sequence, MW or molecular size, and spatial structures are deemed to have a vital role in the activity of APs. In general, APs with a low MW have more opportunities to interact with target molecules to terminate free radical reactions and prevent unsaturated lipid peroxidation [44]. The conclusion was confirmed by the literature that the antioxidant capacities of protein hydrolysates or APs were inversely related to the logarithm of their average MW [29,35]. In addition, small APs have a high likelihood to exploit new drugs because they easily pass through the blood–brain barrier to cause effects in human bodies [32]. The six isolated APs (P1 to P6) are tripeptides to octapeptides with MWs varying from 375.39 to 861.95 Da (Table 1), which shows that they have a high chance of interacting with ROS to prevent lipid peroxidation. 

Previous literature indicated that the kinds of amino acids in the APs were regarded as key factors for their activity [5]. The hydrophobic groups of hydrophobic amino acid residues such as Pro, Met, Ala, Leu, and Ile, can highly react with hydrophobic polyunsaturated fatty acids (PUFAs) to inhibit lipid peroxidation in lipid-rich foods [6,8]. Wu et al. confirmed that the inhibiting ability of Pro-Met-Arg-Gly-Gly-Gly-Gly-Tyr-His-Tyr (PMRGGGGYHY) on free-radical chain reactions was attribute to Met residue because it could serve as a reactive site for formatting a sulfoxide structure to scavenge oxidants [51]. The pyrrolidine ring of the Pro residue can increase the antioxidant ability of Pro-His-His (PHH), Leu-Asp-Glu-Pro-Asp-Pro-Leu (LDEPDPL), Gly-Pro-Gly-Gly-Phe-Ile (GPGGFI), and Phe-Ile-Gly-Pro (FIGP), and to scavenge ROS due to its low ionization potential [52,53]. Zhao et al. [8] and Yang et al. [44] reported that the Ala residues contribute to the ability of Gly-Ile-Glu-Trp-Ala (GIEWA) and Ala-Lys-Gly (AKG) on radical-scavenging and lipid peroxidation inhibition. Moreover, the aromatic groups of Trp, Phe, and Tyr residues can provide protons to stabilize electron-deficient radicals [54,55]. Guo et al. reported that the sequence of APs with Trp residues (Trp-Asn-Glu-His and His-Glu-Trp) and Tyr residues (Arg-Tyr, Lys-Tyr, Tyr-Asp, Tyr-Tyr, Arg-Tyr-Asn, Tyr-Asp-Tyr, Tyr-Glu-Gly, and Tyr-Glu-Glu-Asn) showed high antioxidative ability [54]. The Phe residue in KHNRGDEF can serve as a radical scavenger through providing protons to quench unpaired radicals [55]. In consequence, hydrophobic/aromatic amino acid residues in WMFDW (Trp, Met, and Phe), WMGPY (Trp, Met, and Pro), and EMGPA (Met, Pro and Ala) are vital factors for their activity.

The carboxyl and amino groups in polar amino acid residues are important for the hydroxyl radical scavenging and metal ion chelating capacities of APs [8,30]. Acidic amino acid residues of Asp and Glu were reported to have great influence on the antioxidant activities of Asn-Tyr-Asp-Gly-Ser-Thr-Asp-Tyr-Gly-Ile-Leu-Gln-Ile-Asn-Ser-Arg and Leu-Asp-Glu-Pro-Asp-Pro-Leu-Ile [56,57]. In addition, Gly residue can keep the high flexibleness of the polypeptide skeleton and its single hydrogen atom can be donated to neutralize ROS [5,31,58]. Therefore, polar amino acids, including the Asp residue in WMFDW, Gly residue in WMGPY, and Gly and Glu residues in EMGPA, should play a critical role in their scavenging activities of hydroxyl radicals.

Heating and acid–base treatments are common processing methods in the food industry. APs with heat-resistant properties are beneficial to increase the quality guarantee period of food products when they can retain all or major activity after heating treatment, and APs can be used in more kinds of liquid products if they keep their bioactivity in a wide range of pH values [19]. Therefore, the thermal and pH stability of APs are key properties for designing their specific parameters for application in food products [13,36,59]. Wong et al. prepared two antioxidant hexapeptides (Trp-Ala-Phe-Ala-Pro-Ala and Met-Tyr-Pro-Gly-Leu-Ala) from the hydrolysate of blue-spotted stingray and confirmed that the two hexapeptides had excellent stability because no significant difference was found when they were treated at 25–100 °C or at pH values of 3–11 (*p* > 0.05) [19]. In contrast, Ala-Thr-Ser-His-His from the hydrolysate of sandfish incubated at 50–90 °C reduced its partial DPPH radical scavenging activity. In addition, ATSHH absorbed moderate losses of activity when it was treated at pH 10–12 or pH 2. Yang et al. released homologous conclusions that Met-Asp-Leu-Phe-Thr-Glu and Trp-Pro-Pro-Asp from protein hydrolysates of *Tergillarca granosa* were not appropriate for high-temperature processing (>80 °C) and alkaline conditions (pH > 9.0) in food products [13]. The present results indicate that P2 (WMFDW), P4 (WMGPY), and P6 (EMGPA) have similar thermal and pH stability with ATSHH, MDLFTE and WPPD, because they can significantly decrease antioxidant activity when they are treated under high temperature (80, and 100 °C) and acid (pH 3) and alkali (pH 9) conditions.

## 5. Conclusions

In this experiment, skipjack tuna (*K. pelamis*) heads were hydrolyzed by in vitro GI digestion and six APs separated from the hydrolysate (KPH) were identified as VEE, WMFDW, DAGPYGPI, WMGPY, ERGPLGPH, and EMGPA, respectively. The six isolated APs, especially MDLFTE, WPPD, and EMGPA, exhibited high radical scavenging, reducing power, and lipid peroxidation inhibition capabilities. In addition, MDLFTE, WPPD, and EMGPA are unstable and can keep effective antioxidant activity when environmental temperatures are lower than 60 °C and pH values range from 5 to 9. The investigation showed that the hydrolysates and APs from skipjack tuna (*K. pelamis*) heads can be served as antioxidant functional ingredients in health care products.

## Figures and Tables

**Table 1:** Retention time (RT), molecular mass (Da), and amino acid sequences of six isolated antioxidant peptides (APs) (P1 to P6) from the protein hydrolysate of skipjack tuna heads.

No.	RT (min)	Theoretical Mass/Observed Mass (Da)	Amino Acid Sequence
P1	9.05	375.37/375.39	VEE
P2	11.36	783.89/783.90	WMFDW
P3	12.63	788.84/788.85	DAGPYGPI
P4	13.25	652.76/652.79	WMGPY
P5	16.78	861.94/861.95	ERGPLGPH
P6	17.22	503.57/503.58	EMGPA

**Table 2:** EC_50_ vales of six isolated APs (P1 to P6) on the DPPH radical, hydroxyl radical and superoxide anion radical.

No.	EC_50_ (mg/mL)		
	DPPH Radical	Hydroxyl Radical	Superoxide Anion Radical
P1	3.76 ± 0.16 ^a^	2.43 ± 0.11 ^a^	1.79 ± 0.11 ^a^
P2	0.31 ± 0.02 ^b^	0.30 ± 0.03 ^b^	0.56 ± 0.04 ^b^
P3	1.33 ± 0.09 ^c^	1.71 ± 0.06 ^c^	1.51 ± 0.11 ^c^
P4	0.33 ± 0.02 ^b^	0.43 ± 0.02 ^d^	0.38 ± 0.03 ^d^
P5	0.93 ± 0.04 ^d^	0.81 ± 0.04 ^e^	3.04 ± 0.18 ^e^
P6	0.46 ± 0.04 ^e^	0.52 ± 0.04 ^d^	0.71 ± 0.04 ^b^
GSH	0.22 ± 0.01 ^b^	0.12 ± 0.01 ^f^	0.09 ± 0.01 ^f^

The data are presented as the mean ± SD (*n* = 3). ^a–f^ Values with the same letters of different samples indicate no significant difference (*p* > 0.05).
